# How Can Smoking Cessation Be Induced Before Surgery? A Systematic Review and Meta-Analysis of Behavior Change Techniques and Other Intervention Characteristics

**DOI:** 10.3389/fpsyg.2017.00915

**Published:** 2017-06-07

**Authors:** Andrew Prestwich, Sally Moore, Alwyn Kotze, Luke Budworth, Rebecca Lawton, Ian Kellar

**Affiliations:** ^1^School of Psychology, University of LeedsLeeds, United Kingdom; ^2^Bradford Institute for Health ResearchBradford, United Kingdom; ^3^Department of Anaesthesia, Leeds Teaching Hospitals NHS TrustLeeds, United Kingdom

**Keywords:** smoking, smoking cessation, pre-operative, systematic review, meta-analysis, behavior change technique, mode, intervention

## Abstract

**Background:** Smokers who continue to smoke up to the point of surgery are at increased risk of a range of complications during and following surgery.

**Objective:** To identify whether behavioral and/or pharmacological interventions increase the likelihood that smokers quit prior to elective surgery and which intervention components are associated with larger effects.

**Design:** Systematic review with meta-analysis.

**Data sources:** MEDLINE, Embase, and Embase Classic, CINAHL, CENTRAL.

**Study selection:** Studies testing the effect of smoking reduction interventions delivered at least 24 h before elective surgery were included.

**Study appraisal and synthesis:** Potential studies were independently screened by two people. Data relating to study characteristics and risk of bias were extracted. The effects of the interventions on pre-operative smoking abstinence were estimated using random effects meta-analyses. The association between specific intervention components (behavior change techniques; mode; duration; number of sessions; interventionist) and smoking cessation effect sizes were estimated using meta-regressions.

**Results:** Twenty-two studies comprising 2,992 smokers were included and 19 studies were meta-analyzed. Interventions increased the proportion of smokers who were abstinent or reduced smoking by surgery relative to control: *g* = 0.56, 95% CI 0.32–0.80, with rates nearly double in the intervention (46.2%) relative to the control (24.5%). Interventions that comprised more sessions, delivered face-to-face and by nurses, as well as specific behavior change techniques (providing information on consequence of smoking/cessation; providing information on withdrawal symptoms; goal setting; review of goals; regular monitoring by others; and giving options for additional or later support) were associated with larger effects.

**Conclusion:** Rates of smoking can be halved prior to surgery and a number of intervention characteristics can increase these effects. There was, however, some indication of publication bias meaning the benefits of such interventions may be smaller than estimated.

**Registration:** Prospero 2015: CRD42015024733

## Introduction

Although tobacco use is decreasing [Action on Smoking and Health (ASH), 2016], around one in three patients who undergo elective surgery use tobacco (Kleinwächter et al., 2010; Bradley et al., 2011). This is an important issue because pre-operative smoking can increase the length of hospital stay (Lavernia et al., 1999; Barrera et al., 2005; London Health Observatory, 2016), as well as mortality (based on adjusted risk ratios only), general morbidity, general infections, wound complications, pulmonary complications, neurological complications, and the likelihood of being admitted to intensive care following operation (Grønkjær et al., 2014). However, there is evidence that smoking cessation can be achieved before surgery and that, when attained through intensive, multi-session interventions, there are also reductions in general complications and wound complications (Thomsen et al., 2014).

While Thomsen et al.'s (2014) review demonstrated that both brief and intensive interventions helped to achieve pre-operative smoking cessation with intensive interventions particularly effective, it did not elucidate other active ingredients underlying these increased rates. These characteristics could include those of the person delivering the intervention, the mode of delivery, or the behavior change techniques (BCTs) incorporated.

Taxonomies of behavior change techniques (Michie et al., 2013) comprising standardized descriptions of intervention components have been recently developed, encouraging a systematic approach to intervention development and explicit reporting. Just as biochemists explain the molecular structure of medicines, and their biological mechanisms, behavioral-scientists must define the structure of their interventions, and mechanisms by which they modify behavior. Individual behavior change techniques (BCTs) are the smallest “active ingredients” of a behavioral intervention package that are compatible with retaining a specified mechanism of action (Michie et al., 2015). While the specific BCTs effective in smoking interventions for those attending Stop Smoking Services in England (West et al., 2010) or for those with COPD (Bartlett et al., 2014) have been identified, this is not the case for those awaiting surgery. Moreover, beyond BCTs, group interventions have been linked with higher success rates than one-to-one interventions (Brose et al., 2011) but it is not clear whether this approach would be effective in pre-operative contexts.

There is thus evidence that pre-operative smoking cessation support is needed to improve surgical outcomes (“pre-operative services *should* intervene”), but considerable uncertainty over what the best support is in this setting (“*how* pre-operative services should intervene”). Consequently, the first aim of this review was to characterize the BCTs and other intervention characteristics used in pre-operative intervention studies, and identify, via meta-regression, those associated with higher rates of pre-operative smoking cessation. Second, given the possibility that seemingly effective characteristics, including behavior change techniques, may be confounded with one another (Prestwich et al., 2014, 2016; Peters et al., 2015), we accounted for such potential confounds in the analyses. Third, we extended previous reviews that only incorporated randomized controlled trials (Mills et al., 2011; Thomsen et al., 2014) by also including quasi-experimental trials and single group (pre-post) designs. Fourth, we assessed whether features of study quality and publication bias could impact on the summary estimates, given study quality can bias estimates of effects (Detsky et al., 1992; Prestwich et al., 2014, 2016).

## Methods

The review was registered at Prospero CRD42015024733 and follows the PRISMA reporting guidelines.

### Eligibility criteria

Studies were included if (1) they tested the effect of an intervention (behavioral and/or pharmacological) to reduce smoking; (2) in smokers scheduled for elective surgery and (3) a measure of their smoking in the pre-operative period was taken. Studies were excluded if (1) the intervention was delivered in the intra-operative or post-operative periods only (in particular, the intervention had to begin at least 24 h before surgery); (2) it was a review or commentary; (3) was not published in English; (4) the article was an abstract or dissertation.

### Electronic searches

MEDLINE (1946-) and Embase Classic + Embase (1947-) were searched via OVID. We also searched CINAHL and CENTRAL. The search terms were based on those used by Thomsen et al. (2014) but with study design terms added (Lancaster et al., 2013) to capture non-RCT designs (see Table 1 for our MEDLINE search terms). Where eligible studies referred to associated papers for further methodological, statistical, or intervention-related details, these associated papers were retrieved and used for coding purposes. The searches were last run on the 27th September, 2014.

**Table 1:** MEDLINE search strategy via OVID.

1. Randomized Controlled Trial.pt.
2. Controlled Clinical Trial.pt.
3. Clinical Trial.pt.
4. Exp Clinical trial/
5. Random Allocation/
6. Randomized controlled trials/
7. Double blind method/
8. Single blind method/
9. Placebos/
10. Research Design/
11. (clin$ adj5 trial$ or placebo$ or random$).ti,ab.
12. ((singl$ or doubl$ or trebl$ or tripl$) adj5 (blind$ or mask$)).ti,ab.
13. (volunteer$ or prospectiv$).ti,ab.
14. exp Follow Up Studies/
15. exp Retrospective Studies/
16. exp Prospective Studies/
17. exp Evaluation Studies/or Program Evaluation.mp.
18. exp Cross Sectional Studies/
19. exp Behavior therapy/
20. exp Health Promotion/
21. exp Community Health Services/
22. exp Health Education/
23. exp Health Behavior/
24. 1 or 2 or 3 or 4 or 5 or 6 or 7 or 8 or 9 or 10 or 11 or 12 or 13 or 14 or 15 or 16 or 17 or 18 or 19 or 20 or 21 or 22 or 23
25. smoking cessation.mp. or exp Smoking cessation/
26. “Tobacco-Use-Cessation”/
27. “Tobacco-Use-Disorder”/
28. exp Smoking/pc, th
29. (surgery or operation or operativ: or an?esthesia).mp.
30. exp Postoperative complication/
31. exp Preoperative care/
32. exp Patient education/
33. 30 and (31 or 32)
34. 29 or 33
35. 25 or 26 or 27 or 28
36. 24 and 34 and 35

### Study selection

All records were screened independently by two raters at all stages (titles/abstracts; full-texts). Studies identified as eligible for possible inclusion by either reviewer at the title/abstract stage were included in the full-text screening. Discrepancies were resolved through discussion.

### Data extraction

The lead author coded all of the studies meeting the inclusion/exclusion criteria. To maximize reliability, all elements of data extraction (including effect size calculations) were checked by the last author. Both reviewers were experienced in conducting systematic reviews, had been formally trained in coding behavior change techniques during the development of an extensive list of behavior change techniques (Michie et al., 2013) and were qualified up to PhD level. With the exception of BM10 in the experimental condition (1 disagreement, kappa = 0) and face-to-face delivery in the experimental condition (2 disagreements, kappa = 0.46) all inter-rater reliabilities for the categorical predictors were at least substantial (range: 0.65–1). Disagreements were resolved in consultation with a third reviewer.

The behavior change techniques (BCTs), other aspects of the intervention (duration; number of sessions; mode of delivery; interventionist), participants (type of surgery; country), design, measures, and risk of bias were coded. BCTs were identified in the treatment and comparison groups using a reliable taxonomy comprising descriptions of 44 smoking-specific techniques (Michie et al., 2011). To enable comparison with other reviews and studies, we have labeled the BCTs as recommended by Michie et al. (2011) within the results tables and within the text (e.g., BS9 Set graded tasks represents setting small achievable goals where appropriate (e.g., take 1 day at a time); see Michie et al. (2011) for full definitions of all behavior change techniques). Extra BCTs not covered in this taxonomy (e.g., self-talk) were also coded. Risk of bias was considered using the Cochrane Risk of Bias Tool.

### Data analysis

Effect sizes (*g*) for each study were calculated using Comprehensive Meta-Analysis (Borenstein et al., 2005). Effect sizes were calculated based on the proportion of patients who were abstinent pre-surgery relative to the number of patients randomized to each condition (i.e., an intention-to-treat approach). Where abstinence was not reported, a measure of smoking reduction was used as the basis for the effect size calculation.

STATA version 13.1 (StataCorp, 2013) was used to conduct random-effects meta-analyses and random effects meta-regressions. Meta-analyses examined the extent to which pre-operative smoking cessation can be achieved through intervention. Meta-regressions tested which intervention components and other study features were associated with effect sizes.

Further analyses tested how the results changed when accounting for: trial design (i.e., RCTs only, sensitivity analysis 1); the potential impact of risk of bias (sensitivity analysis 2); confounding between BCTs (sensitivity analysis 3); confounding between BCTs and other intervention characteristics (sensitivity analysis 4); confounding between other intervention characteristics (sensitivity analysis 5). Chi-square analyses were conducted to examine associations between effective intervention components that were assessed categorically (BCTs; interventionist; mode) while Pearson's correlation assessed the associations between these categorical variables and other effective intervention characteristics (duration of intervention; number of sessions). When pairs of BCTs/intervention characteristics where related, they were entered simultaneously as predictors of effect sizes of smoking abstinence in multivariate meta-regressions. If either or both of the BCTs/intervention characteristics were significant in these meta-regressions, this would suggest that the BCTs/intervention characteristics influence smoking abstinence over and above the co-varied feature. If both are non-significant then it is unclear which of the intervention components are effective due to issues of confounding.

The risk of publication bias was assessed using Egger's regression (Egger et al., 1997) and a funnel plot. Duval and Tweedie's (2000) trim and fill analysis estimated the impact of publication bias on effect sizes indicating the effect of the interventions on pre-operative smoking cessation.

## Results

**Figure 1:**
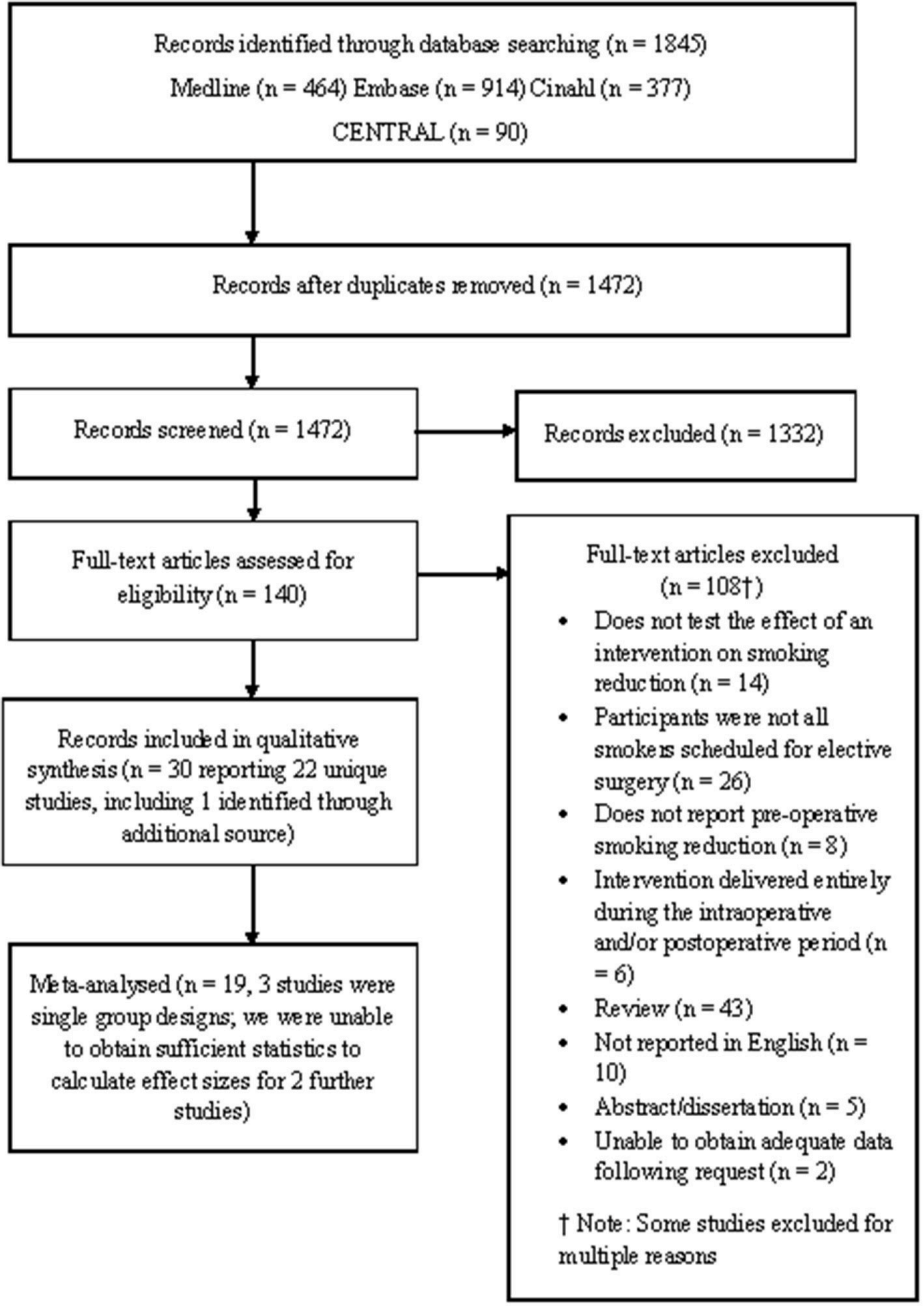
PRISMA Flow Diagram.

Twenty-two studies met the eligibility criteria compared to 13 RCTs included in Thomsen et al.'s (2014) review. Of the 22 eligible studies, 19 were subjected to meta-analysis (three studies used single group pre-post design; see Figure 1). Of these 19 studies, 16 were RCTs and 3 were quasi-experimental studies. Two further studies were identified as eligible but did not report sufficient data in order to calculate effect sizes (Bradley et al., 2013; Hiramatsu et al., 2014). These studies are not discussed further.

### Study characteristics

The studies were conducted in the USA/Canada (*k* = 8), UK (*k* = 6), Denmark/Sweden (*k* = 5), and Australia (*k* = 3) (see Table 2). On average, the interventions were delivered over a period of 28 days (median = 8 days) and involved 2.33 sessions (median = 1; 56% (10/18 studies) used a single session; 4 unclear). The majority of interventions were delivered face-to-face (86%; 19/22) and used written or printed materials (59%; 10/17; 5 unclear). Half of the studies delivered at least part of the intervention using the telephone (50%; 10/20; 2 unclear); fewer studies delivered at least part of the intervention through the internet or computer (19%; 3/16; 6 unclear) or mail (7%; 1/15; 7 unclear). The interventions were delivered by nurses (69%; 11/16; 6 unclear), anesthetists (19%; 3/16), clinicians other than nurses (38%; 6/16), and researchers (19%; 3/16). About a third delivered the intervention through a team of people (31%; 5/16). None of the interventions were delivered to groups of patients. Studies required participants to not smoke for 7 days (mean average) in order to be classified as abstinent (although the median was 1 day).

**Table 2A:** Characteristics of included studies.

**Study**	**Design**	**Scheduled surgery**	**Country**	**BCTs (experimental)**	**BCTs (comparison)**	**%Abstinent**	
						**Expt**.	**Control**
Andrews et al., 2006	RCT	Unclear	UK	BM1b; A5; RI1,2; Ext1	BM1b; A5; RI1,2	35.3	15.7
Haddock and Burrows, 1997	Quasi	General/Gynecology	UK	BM1,9; BS6,7; A2,5; RI1,2,3; RC1,2,7,10; Ext1,8	RI1,2,3;	80.0^a^	50.0^a^
Haile et al., 2002	Single group	Non-cardiac	Australia	BM1,6; BS1,4,7,8; A2; RD1; RI1,2; RC8; Ext1,8,9	n/a	39.3	n/a
Kunzel et al., 2012	Single group	Various	USA	BM1b,2,11;BS4; A5;RI1,2,3	n/a	10.5	n/a
Lee et al., 2013	RCT	Any surgery	Canada	BM1,2,6,8,9,11; A1,2,3,5; BS1,2,3,4,6,7,8,9,10,11; RD1,2; RI1,3; RC:5,6,10; Ext:1,2,4	BM11; RI1	14.3	3.6
Lindström et al., 2008	RCT	Hip/knee/hernia/laparoscopic cholecystectomy	Sweden	BM1,2,11; BS4,5,6b; A3,5; RI1; Ext2	BM11; RI1	36.4	1.6
McHugh et al., 2001	RCT	Coronary artery bypass grafting	UK	BM1,9; A5; RD1; RI1,2; RC8	RI1	92.3	10.0
Møller et al., 2002	RCT	Hip/knee	Denmark	BM1,11; BS4,5,6b; A3; RI1; RC2,6; Ext2,3	RI1	60.0	6.7
Munday et al., 1993	Quasi	Unclear	UK	BM1c; BS4; RI1; Ext1	RI1	7.4	9.3
Myles et al., 2004	RCT	Various	Australia	BM1c;2c,11; BS4; RI1,3; Ext1,2	BM1c,2c,11; BS4; RI1,3; Ext1	4.2	4.3
Ostroff et al., 2014	RCT	Cancer	USA	BM1,2,11; BS1,4,9; A1,3; RD1; RI1,2; RC2; Ext2,5	BM1,2,11; BS1,4; A1,3; RI1,2; Ext2,5	44.8	44.9
Ratner et al., 2004	RCT	Various	Canada	BM1,11; BS4,10; A2,3,5; RI1,2; Ext1,2,6	BM11; RI1,2	69.2	51.7
Shah et al., 1984	Quasi	Various	UK	BS4,6; RI1	none	46.0	14.0
Shi et al., 2013	RCT	Unclear	USA	BM1,2b,11; BS4; RI1,2,3; RC3; Ext1	BM1,2b,11; BS4; RI1,2,3; Ext1	79.3	74.7
Sørensen and Jørgensen, 2003	RCT	Open colonic/rectal procedure	Denmark	BM11; BS4; A3,5; RI1; Ext2	BM11; RI1	80.0^a^	13.3^a^
Sørensen et al., 2007	RCT	Herniotomy	Denmark	BM1c,11; BS4,5; A3; RI1,2; Ext1,2	BM1c,11; BS4; RI1,2; Ext1	19.2	10.0
Thomsen et al., 2010	RCT	Breast cancer	Denmark	BM2,9,11; BS4; RI1,3; Ext5	BM11; RI1,3	24.6	10.8
Walker et al., 2009	Single group	Forefoot osteotomy or arthrodesis	UK	BM1c; BS4; A5; Ext1	n/a	64.0	n/a
Warner et al., 2011	RCT	Various	USA	BM1b; BS4; A5; RI1,3;Ext1	A1,2,5; RI1,3; RC6; BM1,9,10; BS4,8;	6.7	11.9
Warner and Kadimpati, 2012	RCT	Various	USA	BM1b,11; BS3,4; A3; RI1,2,4; Ext2	BM1b,11; BS3,4; RI1,2,4	72.7	54.2
Wolfenden et al., 2005	RCT	Non-cardiac	Australia	BM2; A2,3; RD1; RI1,2,3; Ext2,7	RI1,2,3	74.2	59.3
Wong et al., 2012	RCT	Various	Canada	BM1b,2,11; BS2,4; A3; RI1,2,3; Ext2	BM1b,2,11; BS2,4; RI1,2,3	29.8	20.0

a *Figures represent self-reported rates of reduced smoking. RCT, Randomized Controlled Trial; Quasi, Quasi-experimental trial; single group, Pre-post design with a single condition. Expt, Experimental group. BCTs (Experimental) represent the behavior change techniques delivered to the experimental group. BCTs (Comparison) represent the behavior change techniques delivered to the comparison condition. BCTs are those delivered, at least in part, pre-operation. BCTs delivered exclusively to either the intervention or control groups are underlined. BM1 Provide information on consequences of smoking and smoking cessation; BM2 Boost motivation and self-efficacy; BM6 Prompt commitment from the client there and then; BM9 Identify reasons for wanting and not wanting to stop smoking; BM10 Explain the importance of abrupt cessation; BM11 Measure CO; BS1 Facilitate barrier identification and problem solving; BS2 Facilitate relapse prevention and coping; BS3 Facilitate action planning/develop treatment plan; BS4 Facilitate goal setting; BS5 Prompt review of goals; BS6 Prompt self-recording; BS7 Advise on changing routine; BS8 Advise on environmental restructuring; BS9 Set graded tasks; BS10 Advise on conserving mental resources; BS11 Advise on avoiding social cues for smoking; A1 Advise on stop-smoking medication; A2 Advise on/facilitate use of social support; A3 Adopt appropriate local procedures to enable clients to obtain free medication; A5 Give options for additional and later support; RD1 Tailor interactions appropriately; RD2 Emphasize choice; RI1 Assess current and past smoking behavior; RI2 Assess current readiness and ability to quit; RI3 Assess past history of quit attempts; RI4 Assess withdrawal symptoms; RC1 Build general rapport; RC2 Elicit and answer questions; RC3 Explain the purpose of CO monitoring; RC5 Offer/direct toward appropriate written materials; RC6 Provide information on withdrawal symptoms; RC7 Use reflective listening; RC8 Elicit client views; RC10 Provide reassurance. Additional BCTs (not included within Michie et al. (2011) original taxonomy): BM1b, Variant on BM1 but only refers to the benefits; BM1c, Variant on BM1 but only refers to the harms; BM2b, Variant on BM2 targeting self-efficacy only; BM2c, Variant on BM2 targeting motivation only; BS6b, Variant on BS6 but refers to regular monitoring by others; Ext1, Benefits/harms specified in context of pre-op; Ext2, NRT, Verenicline or Buproprion provided; Ext3, Advise on keeping weight gain to a minimum; Ext4, Self-talk; Ext5, Motivational interviewing; Ext6, BCTs for dealing with cravings and high-risk situations; Ext7, Skills training/practical counseling; Ext8, Directed to NRT but not provided; Ext9: contracts*.

**Table 2B:** Characteristics of included studies.

**Study**	**Delivery (experimental)**				**Delivery (comparison)**			
	**Days**	**Sessions**	**Mode**	**Person delivering**	**Days**	**Sessions**	**Mode**	**Person delivering**
Andrews et al., 2006	1	1	F2F; Print	Nurse; surgeon	1	1	F2F; Print	Nurse
Haddock and Burrows, 1997	10.5	1	F2F; Print	Nurse	1	1	Print	Nurse
Haile et al., 2002	1	1	Computer	n/a	n/a	n/a	n/a	n/a
Kunzel et al., 2012	unclear	2	F2F; Phone; Print	Urologist	n/a	n/a	n/a	n/a
Lee et al., 2013	21	5	F2F; Phone; Print	Nurse + surgeon/anesthetist	Unclear	Unclear	Unclear	Nurse, surgeon, or anesthetist
Lindström et al., 2008	28	4	F2F; Phone	Nurse	Unclear	Unclear	Unclear	Unclear
McHugh et al., 2001	243	8	F2F; Phone	Nurse	243	Unclear	Unclear	Unclear
Møller et al., 2002	49	7	F2F	Nurse	Unclear	Unclear	Unclear	Unclear
Munday et al., 1993	1	1	Print	Unclear	0	0	n/a	n/a
Myles et al., 2004	49	2	F2F; Print	Unclear	49	1	F2F; Print	unclear
Ostroff et al., 2014	7	2	F2F; Computer; Phone; Print	Nurse + researcher	7	2	F2F; Phone	Nurse
Ratner et al., 2004	14	1	F2F; Phone	Nurse	1	1	F2F	Nurse
Shah et al., 1984	1	1	Mail; Print	Unclear	0	0	n/a	n/a
Shi et al., 2013	1	1	F2F	(Anesthetist, internist, physician assistant) + researcher	1	1	F2F	(Anesthetist, internist, physician assistant) + researcher
Sørensen and Jørgensen, 2003	17.5	Unclear	F2F; Phone	Nurse	1	1	F2F	Nurse
Sørensen et al., 2007	Unclear	2	F2F; Phone	Nurse	1	1	Unclear	Unclear
Thomsen et al., 2010	5	1	F2F	Unclear	1	1	F2F	Unclear
Walker et al., 2009	Unclear	Unclear	F2F	Unclear	n/a	n/a	n/a	n/a
Warner et al., 2011	Unclear	Unclear	F2F; Phone; Print	Clinician	Unclear	Unclear	F2F; Print	Clinician
Warner and Kadimpati, 2012	2	1	F2F	Unclear	1	1	F2F	Unclear
Wolfenden et al., 2005	Unclear	Unclear	F2F; Computer	Nurse + anesthetist	Unclear	Unclear	F2F	Nurse + anesthetist
Wong et al., 2012	8	1	F2F; Phone; Print	Researcher	8	1	F2F; Phone; Print	Researcher

*Days/sessions represent the number of days/sessions that participants received behavior change techniques designed to reduce their smoking behavior; mode represents how the behavior change techniques were delivered; F2F, Face-to-face; Person delivering indicates the individual/s who delivered the behavior change techniques; n/a, not applicable; Experimental represents the experimental condition; Comparison represents the comparison condition*.

Aside from the techniques related to measuring smoking behavior or history, the most common BCTs delivered to the intervention group during the pre-operative period were: BS4 Facilitate goal-setting (82%); BM11 Measure carbon monoxide (59%); Ext2 Provide NRT, buproprion or varenicline (50%); A3 Adopt appropriate local procedures to enable clients to obtain free medication (46%); A5 Give options for additional and later support (46%); BM1 Provide information on consequences of smoking and smoking cessation (41%), and BM2 Boost motivation and self-efficacy (32%).

### Can smoking abstinence be achieved pre-operatively?

**Figure 2:**
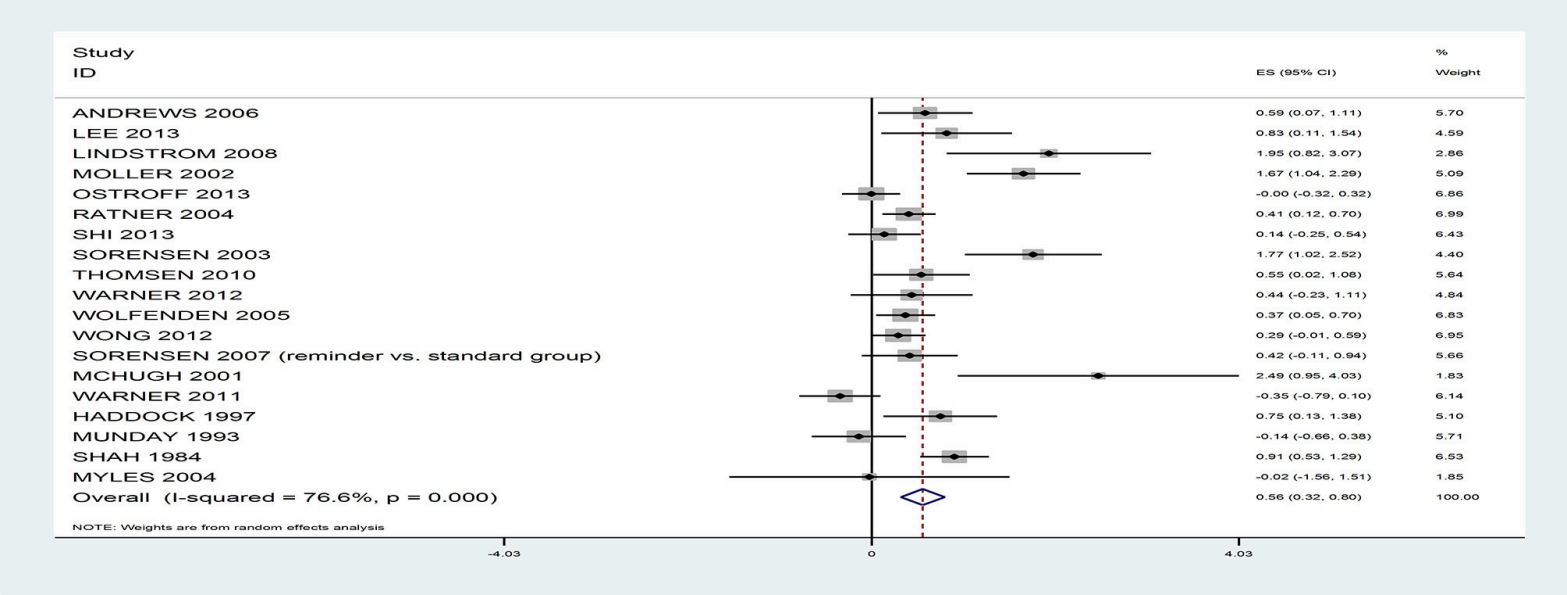
Forest Plot.

The results suggest that rates of smoking reduction can be positively influenced with a medium effect size, *g* = 0.56, 95% CI 0.32–0.80 (see Figure 2), with rates nearly double in the intervention (46.2%) relative to the control (24.5%), based on 19 studies. However, there was significant heterogeneity, *I*^2^ = 76.6%; *Q*_(18)_ = 76.94, *p* < 0.001. When the analyses were repeated only on RCTs (intervention: 46.4% vs. control: 24.5%, based on 16 studies) or quasi-experiments (intervention: 44.5% vs. control: 24.4%, based on 3 studies) or the single group design studies (intervention: 37.9%, based on 3 studies), the rates remained similar.

The majority of study effect sizes reflected rates of abstinence (*k* = 17) rather than reduced smoking (*k* = 2). The overall effect of the interventions on abstinence rates remained medium sized, *g* = 0.48, 95% CI 0.25–0.72 (intervention: 42.1% vs. control: 23.7%, *k* = 17; RCTs only: intervention: 44.2% vs. control: 25.3%, *k* = 15; quasi-experiments only: intervention: 26.7% vs. control: 11.6%, *k* = 2; single group designs: intervention: 37.9%, *k* = 3). From here, the term abstinence is used to refer to the outcomes from all 19 trials. There was an association between the number of days prior to surgery that patients were asked to remain abstinent and the intervention effect sizes; studies that attempted to achieve abstinence further away from the surgery date achieved larger effect sizes, *B* = 0.03, *SE* = 0.01, 95% *CI* 0.007–0.06, *p* = 0.02. The magnitude of the intervention effects did not vary depending on whether the self-reported smoking outcomes were verified biochemically or not, *B* = 0.08, *SE* = 0.32, 95% *CI* −0.58–0.75, *p* = 0.80.

In the single group (pre-post) design studies, the following percentages of participants self-reported that they had stopped smoking prior to surgery: Walker et al. (2009) (16/25; 64%); Haile et al. (2002) (22/56; 39%); Kunzel et al. (2012) (9/38; 28%- but this fell to 13% when biochemically verified).

### Features of interventions that achieve higher rates of abstinence

#### Behaviour change techniques

The following six behavior change techniques were associated with higher rates of smoking abstinence: “Provide information on consequences of smoking and smoking cessation” (BM1); “Facilitate goal setting” (BS4); “Prompt review of goals” (BS5); “Regular monitoring by others” (BS6b); “Give options for additional and later support” (A5); and, “Provide information on withdrawal symptoms” (RC6) (Table 3). However, “Facilitate goal setting” (BS4) only reached conventional levels of significance when the analyses were based only on the RCTs and “Prompt review of goals” (BS5) was only marginally significant when either based on all trials or only on RCTs. From the studies that employed a single group (pre-post) design, it is interesting to note that the study that reported the highest rate of abstinence (Walker et al., 2009), few BCTs were employed but, of these, two were BCTs identified as being effective in the experimental trials [“Facilitate goal setting” (BS4); “Give options for additional and later support pre-surgery” (A5)].

**Table 3:** Meta-regressions.

**Predictor**	**Expt. group only**	**Both groups/neither**	**Cont. group only**	**RCTs** + **Quasi (*****k*** = **19)**				**RCTs only (*****k*** = **16)**			
				**B**	**95% CI**		***p*-value**	**B**	**95% CI**		***p*-value**
					**Lower limit**	**Upper limit**			**Lower limit**	**Upper limit**	
BM1Information on smoking/cessation consequences	6	12	1	0.69	0.21	1.17	0.01^**^	0.75	0.22	1.28	0.01^**^
BM2 Boost motivation/self-efficacy	4	15	0	0.25	−0.56	1.07	0.52	0.25	−0.66	1.15	0.57
BM9 Identify reasons for wanting/not wanting to stop	4	14	1	0.51	−0.13	1.15	0.11	0.59	−0.17	1.34	0.12
BS4 Goal setting	8	11	0	0.54	−0.05	1.14	0.07^†^	0.77	0.13	1.40	0.02^*^
BS5 Review of goals	3	16	0	0.75	−0.11	1.61	0.08^†^	0.76	−0.16	1.69	0.10^†^
BS6 Self-recording	3	16	0	0.30	−0.58	1.18	0.48	0.23	−1.44	1.89	0.78
BS6b Regular monitoring by others	2	17	0	1.32	0.34	2.29	0.01^*^	1.33	0.32	2.35	0.01^*^
BS7 Changing routine	2	17	0	0.22	−0.87	1.32	0.67	0.23	−1.44	1.89	0.78
BS8 Environmental restructuring	1	17	1	0.63	−0.31	1.57	0.18	0.63	−0.38	1.64	0.20
BS9 Set graded tasks	2	17	0	−0.27	−1.31	0.78	0.60	−0.29	−1.44	0.85	0.59
BS10 Conserving mental resources	2	17	0	−0.00	−1.05	1.05	0.99	−0.03	−1.18	1.25	0.96
A1 Advise on stop-smoking medication	1	17	1	0.63	−0.31	1.57	0.18	0.63	−0.38	1.64	0.20
A2 Use of social support	4	14	1	0.19	−0.45	0.84	0.53	0.18	−0.58	0.94	0.62
A3 Enable clients to obtain free medication	9	10	0	0.43	−0.19	1.04	0.16	0.50	−0.23	1.23	0.17
A5 Give options for extra support	6	13	0	0.74	0.11	1.38	0.02^*^	0.85	0.13	1.58	0.03^*^
RD1 Tailor interactions	4	15	0	0.02	−0.81	0.85	0.96	0.02	−0.91	0.94	0.97
RC2 Elicit and answer questions	3	16	0	0.19	−0.69	1.07	0.65	0.17	−0.97	1.30	0.76
RC6 Information on withdrawal symptoms	2	16	1	0.80	0.12	1.48	0.02^*^	0.80	0.09	1.51	0.03^*^
RC10 Provide reassurance	2	17	0	0.22	−0.87	1.32	0.67	0.23	−1.44	1.89	0.78
Ext1 Benefits/harms specified in context of pre-op	6	13	0	−0.39	−1.06	0.28	0.23	−0.37	−1.22	0.49	0.37
Ext2 NRT, Verenicline or Buproprion provided	10	9	0	0.37	−0.25	1.00	0.22	0.44	−0.31	1.19	0.23

* p < 0.05;

** p < 0.01;

† *p < 0.10*.

*Only the behavior change techniques that are used exclusively in one condition for at least 2 studies are reported. k,number of studies; RCTs, Randomized controlled trials; Quasi, Quasi Experimental Trials; Expt. Group only indicates the number of studies that employed the behavior change technique only in the Experimental group; Both groups/Neither indicates the number of studies that employed the behavior change technique in both experimental and comparison groups, or in neither; Cont. group only indicates the number of studies that employed the behavior change technique only in the comparison condition. B, regression coefficient; 95%CI = 95% confidence interval*.

*Behavior change techniques regressed on smoking cessation effect sizes*.

#### Other intervention characteristics

Longer delivery and more intervention sessions for the experimental group yielded larger effect sizes, as did face-to-face delivery, using modes of delivery other than print, and delivery of the intervention by a nurse (Table 4). Of the studies that used a single group (pre-post) design, Haile et al.'s (2002) intervention was delivered via a computer (and achieved 39.3% abstinence), while Walker et al. (2009) who reported a much higher rate of abstinence (64.0%) was delivered face-to-face. Although Kunzel et al. (2012), who reported much lower rates of abstinence (10.5%) also delivered their intervention face-to-face, they also used telephone delivery and print-based materials.

**Table 4:** Meta-regressions.

**Study characteristic**	***k***	**B**	**RCTs** + **Quasi (*****k*** = **19) 95% CI**		**RCTs only (*****k*** = **16)**	
			**Lower limit**	**Upper limit**	***p*-value**	***p*-value**
**INTENSITY**						
Duration of delivery (intervention group, days)	16	0.01	0.002	0.019	0.02^*^	0.03^*^
Duration of intervention (*relative* to comparison group)	13	0.04	−0.02	0.10	0.13	0.17
Number of sessions (intervention group)	16	0.22	0.09	0.34	0.002^**^	0.001^**^
Number of sessions (*relative* to comparison group)	12	0.07	−0.42	0.57	0.75	0.80
**MODE OF DELIVERY TO THE INTERVENTION GROUP**						
Face-to-face	19	0.21	−0.82	1.23	0.68	−
Internet/computer	19	−0.46	−1.42	0.50	0.32	0.32
Telephone	19	0.09	−0.58	0.76	0.78	0.80
Mail	19	0.34	−1.02	1.70	0.60	−
Print	19	−0.50	−1.11	0.10	0.099^†^	0.09^†^
**MODE^a^**						
Face-to-face	19	0.77	0.14	1.39	0.02^*^	0.02^*^
Internet/computer	19	−0.46	−1.42	0.50	0.32	0.32
Telephone	19	0.36	−0.33	1.04	0.29	0.30
Mail	19	0.34	−1.02	1.70	0.60	−
Print	19	−0.27	−1.04	0.51	0.47	0.59
**INTERVENTIONIST FOR THE EXPERIMENTAL CONDITION^b^**						
Nurse	19	0.58	−0.01	1.17	0.05^†^	0.06
Anesthetist	19	−0.21	−1.08	0.66	0.62	0.59
Team	19	−0.32	−1.03	0.38	0.35	0.31
Clinician (doctor, anesthetist, surgeon, urologist)	19	−0.41	−1.10	0.28	0.23	0.20
**INTERVENTIONIST^c^^,^^d^**						
Nurse	19	0.95	0.20	1.69	0.02^*^	0.02^*^
Team	19	−0.32	−1.03	0.38	0.35	0.63

*Study characteristics other than behavior change techniques regressed on smoking cessation effect sizes*.

* p < 0.05;

** p < 0.01;

† *p < 0.10*.

k, number of studies; B, regression coefficient; 95%CI = 95% confidence interval; RCTs, randomized controlled trials; Quasi, Quasi-Experimental Trials;

a *Reflects the specific mode of delivery in the intervention group controlling for the equivalent mode in the comparison group. This was achieved by coding mode present as +1 and mode absent as 0 and then subtracting the mode of delivery in the comparison group from the mode of delivery in the intervention group. Thus, +1 reflects the intervention group only used the specific mode; 0 reflects either both the intervention and comparison groups employed that mode, or neither; −1 reflects the comparison group only used the specific mode of delivery*.

b *Repeating these analyses after excluding the studies in which it was unclear who delivered the intervention did not significantly influence the findings (i.e., the marginal effect remained marginal and the non-significant effects remained non-significant)*.

c *Adopts an equivalent approach to ^a^*.

d *Results not reported for anesthetist as there were no studies in which the anesthetist delivered the intervention component without delivering the comparison component. Results not reported for clinician as only one study used a clinician to deliver the intervention without using a clinician to deliver the comparison component*.

### Sensitivity analyses

#### Sensitivity analyses 1: randomized controlled trials only

The effects of the BCTs on smoking abstinence effect sizes were largely unaffected by whether or not the study design was a randomized controlled trial (*k* = 16). Specifically, all of the significant BCTs remained significant and the non-significant BCTs remained non-significant. The only exception was “Goal setting” (BS4) which became significant rather than marginally significant (Table 3).

#### Sensitivity analyses 2: does risk of bias influence effect sizes?

The studies were at variable risk of bias (Table 5). For example, most studies were at low risk of bias from inadequate randomization but none of the studies were at low risk of bias from selective reporting. Potential methodological quality confounds were examined by establishing whether the risk of bias indices were associated with effect size. None of the risk of bias indices (randomization, allocation concealment, blinding, selective outcome reporting, or incomplete data) were associated with effect sizes. This suggests the intervention characteristics associated with higher rates of smoking abstinence were unlikely to have been influenced through differential use within methodologically better (or poorer) studies.

**Table 5:** Risk of Bias.

**Study**	**Adequate randomization?**	**Adequate allocation concealment?**	**Adequate blinding during pre-op?**	**Incomplete outcome data addressed?**	**Free from selective outcome reporting?**
Andrews et al., 2006	Yes	Yes	Unclear	Yes	No
Haddock and Burrows, 1997	No	No	I/DC-No; P/DA-Unclear	Yes	Unclear
Haile et al., 2002,	n/a	n/a	n/a	Unclear	Unclear
Kunzel et al., 2012	n/a	n/a	n/a	Unclear	Unclear
Lee et al., 2013	Yes	Yes	I-No; P/DC/DA-Unclear	Yes	No
Lindström et al., 2008	Yes	Yes	P/I/DA-No; DC-Unclear	Yes	Unclear
McHugh et al., 2001	Unclear	Unclear	P/I/DA-Unclear; DC-No	Unclear	Unclear
Møller et al., 2002	Yes	Yes	P/I/DA-No; DC-Unclear	Yes	Unclear
Munday et al., 1993	No	No	I/DC-No; P/DA-Unclear	Unclear	Unclear
Myles et al., 2004	Yes	Unclear	P/I-Yes;DC-Unclear;DA-No	No	Unclear
Ostroff et al., 2014	Yes	Unclear	Unclear	Yes	Unclear
Ratner et al., 2004	Yes	Yes	P/I/DA-No; DC-Unclear	Unclear	Unclear
Shah et al., 1984	No	No	I/DC-No; P/DA-Unclear	Yes	Unclear
Shi et al., 2013	Unclear	Unclear	No	Unclear	Unclear
Sørensen and Jørgensen, 2003	Yes	Yes	P/I/DA-No; DC-Unclear	Unclear	Unclear
Sørensen et al., 2007	Yes	Yes	No	Unclear	Unclear
Thomsen et al., 2010	Yes	Yes	P/I/DA-No; DC-Unclear	Unclear	Unclear
Walker et al., 2009	n/a	n/a	n/a	Unclear	Unclear
Warner et al., 2011	Yes	Yes	No	Yes	Unclear
Warner and Kadimpati, 2012	Yes	Yes	P/I-Yes; DC/DA-Unclear	Yes	Unclear
Wolfenden et al., 2005	Yes	Unclear	No	Unclear	Unclear
Wong et al., 2012	Yes	Yes	P/I/DC-Yes; DA-Unclear	Yes	No

*P/I/DC/DA, Participant/Interventionist/Data Collector/Data Analyst; n/a, not applicable*.

#### Sensitivity analyses 3: are the effects of the BCTs on smoking abstinence effect sizes confounded by other BCTs?

“Goal setting” (BS4) was delivered independently of the other BCTs so its effects on smoking abstinence appeared not to have been confounded by other BCTs. “Regular monitoring by others” (BS6b) was only related to “Review of goals” (BS5) but it still marginally predicted smoking abstinence, *B* = 1.35, *SE* = 0.70, 95%CI-0.15–2.84, *p* = 0.07, when controlling for BS5. However, “Provide information on smoking/cessation consequences” (BM1) no longer predicted smoking abstinence when controlling for either “Give options for extra support” (A5) or “Provide information on withdrawal symptoms” (RC6). Similarly, “Review of goals” (BS5) and “Provide information on withdrawal symptoms” (RC6) were both non-significant when controlling for “Provide information on smoking/cessation consequences” (BM1). Associations between the variables are reported in Table 6.

**Table 6:** Significance of associations (*p*-values) between predictors of smoking cessation effect sizes.

**Predictor**	**1**	**2**	**3**	**4**	**5**	**6**	**7**	**8**	**9**	**10**	**11**	**12**
1. BM1: info. on smoking/cessation consequences	–	0.27	0.35	0.09^†^	0.004^**^	0.000^**^	0.09^†^	0.006^**^	0.45	0.004^**^	0.03^*^	0.11
2. BS4: goal setting		–	0.55	0.16	0.32	0.16	0.45	0.54	0.65	1.00	1.00	1.00
3. BS5: Review of goals			–	0.02^*^	1.00	0.35	0.81	0.12	0.21	0.02^*^	0.23	0.004^**^
4. BS6b: regular monitoring by others				–	1.00	0.15	0.81	0.04^*^	0.47	0.09^†^	0.49	0.04^*^
5. A5: give options for extra support					–	0.68	0.17	0.11	0.63	0.05^*^	0.02^*^	0.37
6. RC6: Information on withdrawal symptoms						–	0.88	0.01^*^	0.55	0.08^†^	0.24	0.52
7. Duration of delivery to intervention group							–	0.001^**^	0.29	0.05^†^	0.27	0.006^**^
8. Number of sessions for those in the intervention								–	0.25	0.002^**^	0.04^*^	0.001^**^
9. Intervention uses print materials									–	0.63	0.37	0.09^†^
10. Face-to-face intervention vs. not in the comparison										–	0.02^*^	0.004^**^
11. Nurse delivered the intervention											–	0.10
12. Nurse delivered intervention vs. not in the comparison												–

*Fisher's Exact Test p-value reported, where appropriate*.

* p < 0.05;

** p < 0.01;

† *p < 0.10*.

#### Sensitivity analyses 4: are the effects of BCTs on smoking abstinence effect sizes confounded by other important intervention characteristics?

“Goal setting” (BS4) was not related to any feature of the intervention that was significantly related with smoking abstinence effect sizes. The other effective BCTs were related to some extent to other intervention characteristics (see Table 6) that were predictive of smoking abstinence effect sizes (see Table 4). In multivariate meta-regressions, “Provide information on smoking/cessation consequences” (BM1) became a marginally significant predictor of smoking abstinence effect sizes when controlling for nurse delivery, *B* = 0.59, *SE* = 0.28, 95%CI-0.008–1.19, *p* = 0.05. However, all of the other BCTs [aside from “Goal setting” (BS4)] were rendered non-significant when controlling for other relevant intervention characteristics (i.e., those that predict effect sizes and were also associated with the BCT).

#### Sensitivity analyses 5: are the effects of other intervention characteristics on smoking abstinence effect sizes confounded?

The benefit of using other modes of delivery instead of print is unlikely to be confounded by other intervention characteristics given these variables were unrelated (see Table 6). Intervention characteristics, other than BCTs, that were associated with smoking abstinence effect sizes were otherwise inter-correlated. Of these variables, the number of sessions delivered to the experimental group remained predictive of smoking abstinence effect sizes after controlling for delivery of the intervention by the nurse, *B* = 0.22, *SE* = 0.07, 95% CI 0.07–0.36, *p* = 0.006, delivery of the intervention by the nurse relative to the comparison group, *B* = 0.17, *SE* = 0.08, 95% CI 0.00–0.34, *p* = 0.049, and marginally significant when controlling for duration of intervention, *B* = 0.17, *SE* = 0.08, 95% CI-0.01–0.34, *p* = 0.06, and face-to-face delivery, *B* = 0.17, *SE* = 0.08, 95% CI-0.01–0.34, *p* = 0.06. Nurse delivery in the experimental group rather than the comparison group significantly predicted smoking abstinence effect sizes after controlling for delivery duration, *B* = 1.13, *SE* = 0.48, 95%CI 0.08–2.17, *p* = 0.04. No other predictors were significant in this set of sensitivity analyses.

### Publication bias

**Figure 3:**
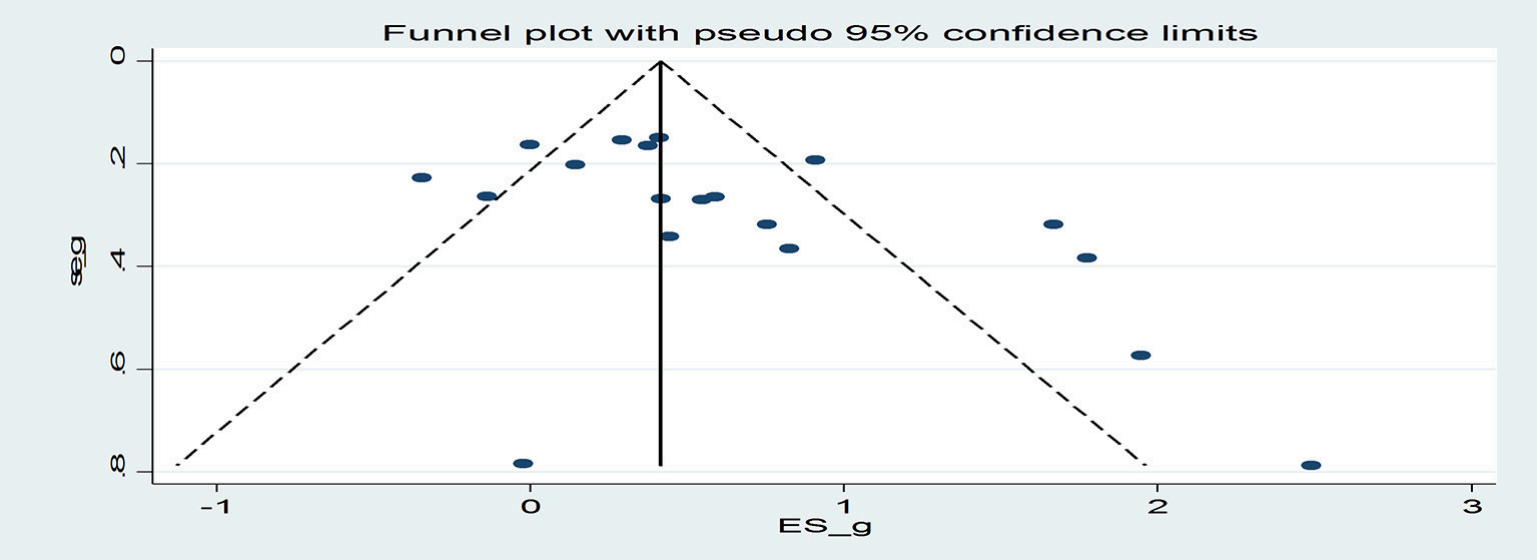
Funnel Plot.

Egger's regression coefficient was significant suggesting some evidence of publication bias (*p* = 0.03; Figure 3). Duval and Tweedie's (2000) trim and fill analysis imputed 6 additional effect sizes for the effect of the interventions on smoking outcomes, resulting in an overall effect size of *g* = 0.26 (CI = −0.004−0.53). Thus, after the trim and fill analysis was conducted, the effect of the interventions on smoking was approximately halved and became marginally significant (*p* = 0.053).

## Discussion

Our findings are consistent with a previous systematic review by Thomsen et al. (2014) and show that patients can effectively be supported in stopping smoking before surgery. In 19 studies involving randomized and quasi-experimental designs, the effect of the interventions was to reduce smoking prevalence by half at the time of surgery (effect size *g* = 0.56, 95% CI 0.32–0.80). The results from the meta-analyses are unlikely to have been influenced by risk of bias across the studies because the risk of bias measures were unrelated to intervention effect sizes. The size of the overall effect was also similar when the quasi-experimental trials were excluded. In addition, we found that cessation support may be optimized with the incorporation of specific intervention components.

### Behaviour change techniques

Of the BCTs, “provide information about the consequences of smoking and smoking cessation” (BM1), was the most significant predictor of quitting successfully. This has significant implications for pre-operative smoking cessation support. The benefits of stopping smoking before surgery are substantially different, and may be realized sooner, than the benefits from smoking cessation in patients who are not scheduled to have operations. Surveys of smokers' knowledge suggest that they are aware of the general health risks of smoking, but under-appreciate that there are specific peri-operative risks (Webb et al., 2013; Bottorff et al., 2015). However, when controlling for other BCTs which were often delivered alongside BM1, the effects drifted to non-significance. Given the number of studies included in the review, and consequent low power for such multivariate meta-analyses, these null findings are perhaps unsurprising. However, it does highlight the issue of potential confounding and indicates that effects, at least in some instances, are not neatly attributed to a single intervention component but potentially to a group of intervention components.

Goal-setting was used frequently, was not confounded with other BCTs or intervention features, and was positively associated with effect size. This suggests that goal-setting appears to be a promising BCT to help smokers to abstain from smoking up to the point of surgery with its effects not attributable to other BCTs or intervention features.

Regular monitoring by others (BS6b) was also associated with larger effect sizes and was marginally significant when controlling for other related BCTs. However, *regular* monitoring appears to be essential in light of the results from Shi et al.'s (2013) study which indicated that informing participants that their smoking intake would be monitored once on the day of surgery did not increase abstinence rates relative to an otherwise equivalent comparison group. Other BCTs that were initially associated with effect sizes became non-significant when controlling for related BCTs.

### Intervention intensity, duration, and mode of delivery

Thomsen et al.'s (2014) review suggested that intensive interventions over multiple sessions were more successful than brief interventions in aiding smoking cessation prior to surgery. Taking a more fine-grained approach to the analyses, our review identified a number of specific intervention components reflecting a more intensive, longer-term approach to support abstinence that were associated with higher rates of pre-operative smoking cessation. In particular, delivering the interventions over more sessions and for a longer duration, using regular monitoring by others (BS6b), giving options for additional and later support (A5), and using face-to-face delivery led to higher rates of abstinence. The use of print materials (e.g., provision of leaflets), which may be indicative of a lower intensity intervention, was *not* helpful in trials to date. Despite the relatively low power within the multivariate meta-regressions, studies that delivered more sessions generated larger effects that were robust when controlling for several other significant intervention components including duration of delivery.

#### Personnel delivering the intervention

Of the interventionists, nurses were used most often and appeared to be the most successful. This could be attributable not only to their status as a credible source of information but also reflect that they may have more time to deliver intensive interventions relative to other healthcare professionals. Indeed, nurses delivered interventions with more sessions and, after controlling for the number of sessions, nurse-based interventions were no longer associated with effect sizes. This suggests that there is a trade-off to be made in busy pre-operative services that aim to present patients for surgery within specified time frames such as 18 weeks in the UK (NHS Clinical Services Team, 2015). Nurses (and/or potentially others) delivering more sessions should be more effective in increasing smoking abstinence in the lead up to surgery but this may be difficult due to time constraints.

### Important gaps in the literature

As well as highlighting intervention components that are potentially effective in promoting smoking cessation in patients about to undergo surgery, the review also highlights gaps in the literature. For instance, a number of behavior change techniques have not been tested in this context such as providing rewards contingent on effort or progress and no interventions were delivered to groups. These areas offer potential because of evidence that providing contingent rewards can be effective in reducing smoking (Giles et al., 2014) and smoking-based interventions delivered to groups have been found to be more effective than those delivered to individuals (Bartlett et al., 2014).

### Limitations

There were a number of limitations that should be discussed. First, by not considering unpublished studies, the estimated effects in this review may be overestimates, because published articles may be more likely to report significant effects than non-published articles. Indeed, relevant analyses suggested there was some evidence of publication bias which, when accounted for, the estimated effect size was halved. However, given unpublished studies had not been peer reviewed and could contain insufficient information, we anticipated that attempts to code BCTs and other intervention content would be unreliable. There is also the possibility that there may be differences between the unpublished data/studies that authors were willing to share and those studies for which authors were not willing to share.

Second, there were relatively few studies meaning several BCTs were used only in one study or not at all. The number of studies analyzed also hindered the multivariate meta-regressions. However, the risk of confounding remains an important issue.

Third, the review could have been conducted using a broader range of databases meaning there is a possibility that some studies were missed. However, the review incorporates more studies (22 studies) than those included in a recent Cochrane review (Thomsen et al., 2014: 13 studies) and employed double-screening which minimizes the risk that studies were excluded in error. Moreover, it incorporated single group designs that yielded results with some similarity with those generated from the experimental trials, highlighting the possible effectiveness of goal setting, providing additional support, and face-to-face delivery.

Fourth, there is possibility of coding errors but coding was checked by a second reviewer and we also coded intervention components within the comparison conditions and took account of this in our analyses. This has not always been done within reviews attempting to identify which BCTs are effective in changing health behaviors and related outcomes (Michie et al., 2009; Dombrowski et al., 2012).

Finally, the average amount of time that patients were required to be abstinent across the studies in this review was 7 days which, in this setting, is perhaps understandable. However, this is a very short period of time for smoking cessation. Indeed, none of the studies included in this review that required smoking cessation for 7 days or less detected significant differences between the intervention and control conditions on rates of post-operative complications; cessation over the longer-term seems to be required to reduce post-operative complications (Møller et al., 2002; Lindström et al., 2008). Given the focus of the review on strategies to promote pre-operative smoking cessation rates, we are unable to offer clear suggestions regarding what should be done post-surgery with patients to promote cessation. However, there is evidence that pre-operative smoking cessation predicts cessation longer-term (Lee et al., 2015) thus achieving brief cessation pre-surgery may be enough, for some patients, to not smoke post-surgery. Interestingly, in an unpublished service evaluation that we have conducted, interviewed patients suggested that they wanted the emphasis to be on cessation during the pre-operative period and not the longer term. Thus, while one of the potential benefits of helping patients to abstain from smoking during the pre-operative period is that they are more likely to quit long-term, emphasizing longer-term abstinence at the onset of the intervention may actually lower engagement.

## Conclusion

The studies included in the review suggest that the percentage of smokers who continue to smoke up until the point of surgery can be halved, reflective of a medium effect size. However, there was a risk of publication bias meaning the overall effect may be smaller. Various intervention components were associated with larger intervention effects but many of these are potentially confounded. However, delivering more sessions, goal setting and, to a lesser extent, regular monitoring of smoking by others were robust against such confounds. Some intervention components shown to be effective in reducing smoking in other populations, such as delivering smoking cessation materials to groups, have not been tested in patients awaiting surgery and could be usefully tested by future research.

## Author contributions

Conceptualization: AP, IK, RL, AK, SM. Formal Analysis: AP, IK. Funding Acquisition: RL, IK, AP, AK, SM. Methodology: AP, IK. Investigation: AP, IK, LB, SM. Writing: Original draft preparation: AP, LB, AK. Writing: Review & Editing: AP, IK, RL, SM, LB, AK.

### Conflict of interest statement

The authors declare that the research was conducted in the absence of any commercial or financial relationships that could be construed as a potential conflict of interest.
